# A Rare Encounter: Mammary-Like Gland Tissue in an Anal Polyp

**DOI:** 10.7759/cureus.103410

**Published:** 2026-02-11

**Authors:** Laci-Rae Pitter, Sania L Siddiqui, Daniel Tomer, Jeffrey Snow, David Marshall, Gary Schwartz

**Affiliations:** 1 College of Allopathic Medicine, Nova Southeastern University Dr. Kiran C. Patel College of Allopathic Medicine, Fort Lauderdale, USA; 2 Internal Medicine, Nova Southeastern University Dr. Kiran C. Patel College of Allopathic Medicine, Fort Lauderdale, USA; 3 Colorectal Surgery, Memorial Healthcare System, Hollywood, USA; 4 Pathology, Memorial Healthcare System, Hollywood, USA; 5 Orthopaedic Surgery, Nova Southeastern University Dr. Kiran C. Patel College of Allopathic Medicine, Fort Lauderdale, USA

**Keywords:** anal polyp, apocrine metaplasia, ectopic breast tissue, estrogen receptor (er), fibrocystic changes, gata3, intraductal papilloma, mammary-like anogenital glands, p63

## Abstract

Anogenital mammary-like glands (AGMLGs) are normal structures in the anogenital region that can undergo pathologic changes similar to breast tissue. This case report presents a 54-year-old female with a complaint of worsening anorectal pain. Her initial physical exam revealed a 1 cm anal polyp. Pathology confirmed breast-like polypoid tissue with benign features, including intraductal papilloma, apocrine metaplasia, and fibrocystic change. The novelty of this case highlights that mammary-like glands can be found outside of the mammary line and may mimic lesions identical to counterparts in the native female breast, underlining that confirmation of diagnosis with histologic evaluation is crucial for management of rare benign and potentially malignant pathologies. Benign lesions can be conservatively excised or monitored based on symptoms and patient preference.

## Introduction

First described in the late 19th century, anogenital mammary-like glands (AGMLG) demonstrate morphologic and immunohistochemical similarities to mammary tissue. Earlier theories proposed that such tissue represented ectopic remnants of the embryologic milk line [1]. This ridge extends bilaterally from the axilla to the inguinal region and typically regresses except at the thoracic segment, where the normal breast develops [2]. According to this view, mammary gland development begins around the sixth week of embryogenesis, when paired linear thickenings extend from the axilla to the groin. By the second to third month of gestation, these thickenings give rise to mammary ridges, from which the normal breasts develop. Incomplete regression of these ridges was thought to result in accessory breast tissue, presenting as additional breasts (polymastia) or nipples (polythelia). These supernumerary structures can arise from extramammary buds or from displaced mammary tissue along the ridge [3].

Ectopic breast tissue has also been reported in unusual sites, including the eyelid, nasal area, prostate gland, and gallbladder [4]. Given that these findings challenge the milk line hypothesis, Van der Putte suggested an alternate theory: anogenital mammary-like glands represent a normal component of the anogenital region [5]. These glands are thought to originate from eccrine-type sweat structures rather than from displaced mammary tissue. They are most frequently identified in the vulva but have also been reported in association with the perineum, anus, and even the male genitalia. They are often recognized only on histopathologic examinations unless they form a discrete mass [4].

AGMLG has been estimated to occur in 1 to 6 percent of the general population and may follow a hereditary pattern. The majority of cases are single and unilateral, although a third of patients may have more than two accessory structures. Though often clinically silent, AGMLG are subject to the same hormonal influences and pathologic changes as orthotopic breast tissue. They may respond to physiologic fluctuations during the menstrual cycle, pregnancy, or lactation and can undergo benign or malignant transformation. Histologically, ectopic breast tissue shows large ducts with prominent stroma and may lack fully developed terminal ductal lobular units [6].

Importantly, anogenital mammary-like glands can give rise to a wide range of lesions that closely resemble those of the breast, including benign entities such as fibroadenoma, lactating adenoma, and hidradenoma papilliferum, as well as malignant processes such as hidradenocarcinoma papilliferum, phyllodes tumor, and invasive carcinoma. Other reported changes include fibrocystic changes, intraductal papilloma, sclerosing adenosis, pseudoangiomatous stromal hyperplasia, atypical ductal hyperplasia, and columnar cell alterations [1,6].

Most cases of AGMLG are discovered incidentally or when hormonal stimulation leads to swelling, pain, or palpable mass formation. In some instances, they may be misdiagnosed as soft tissue tumors, abscesses, or anal polyps. Given the diagnostic challenge and the potential for malignant transformation, histologic confirmation is essential. We present a rare case of breast tissue located in the anorectal region, discovered on histopathologic examination of a polypoid anal lesion. To our knowledge, this is one of the very few reports documenting intraductal papilloma arising outside of the mammary line.

## Case presentation

**Figure 1:**
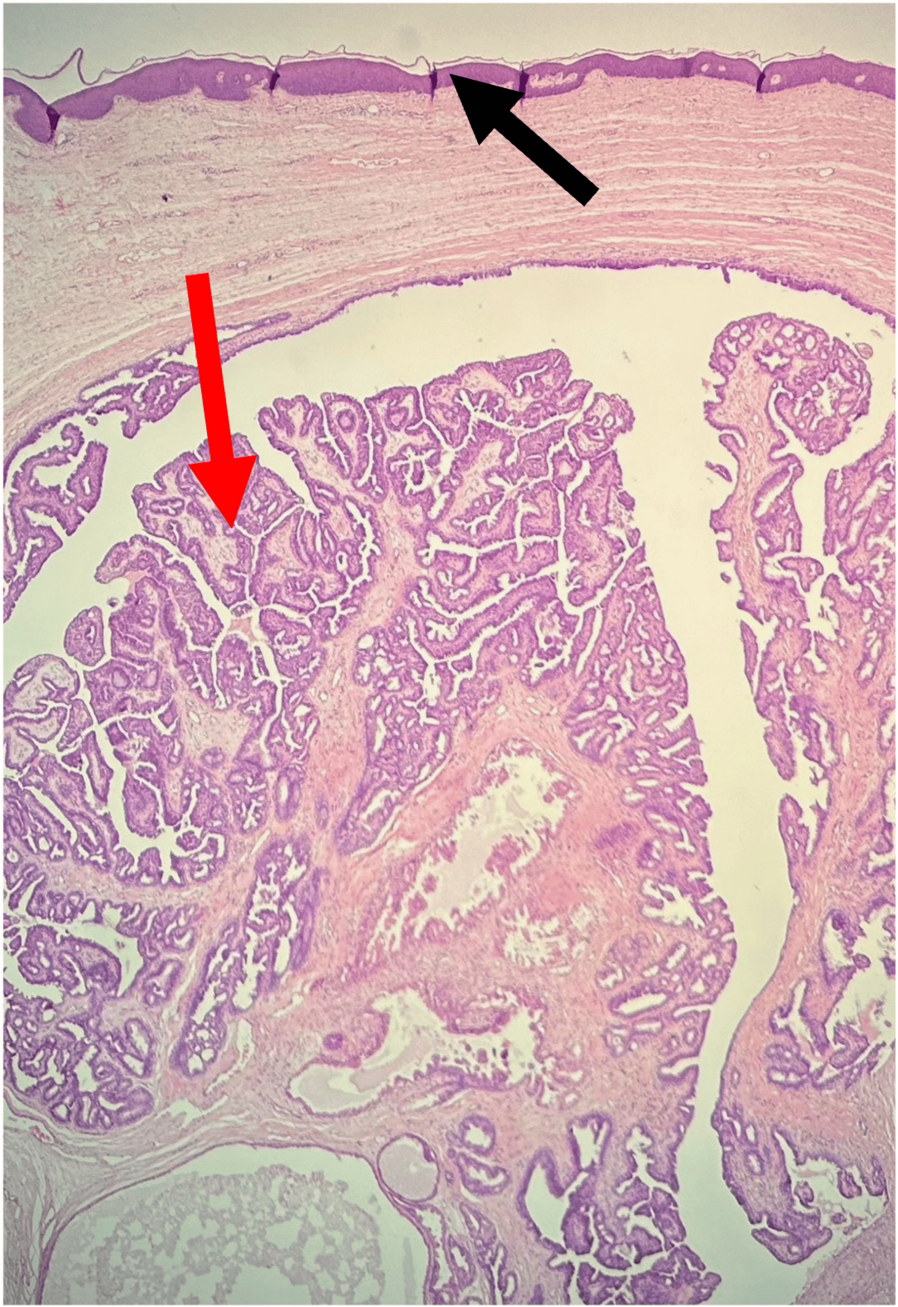
Histopathological findings Low power of the polyp (×2.5) demonstrating a benign tumor of modified anogenital mammary-like glands. The red arrow demonstrates the papillary tumor. The black arrow demonstrates the overlying skin.

**Figure 2:**
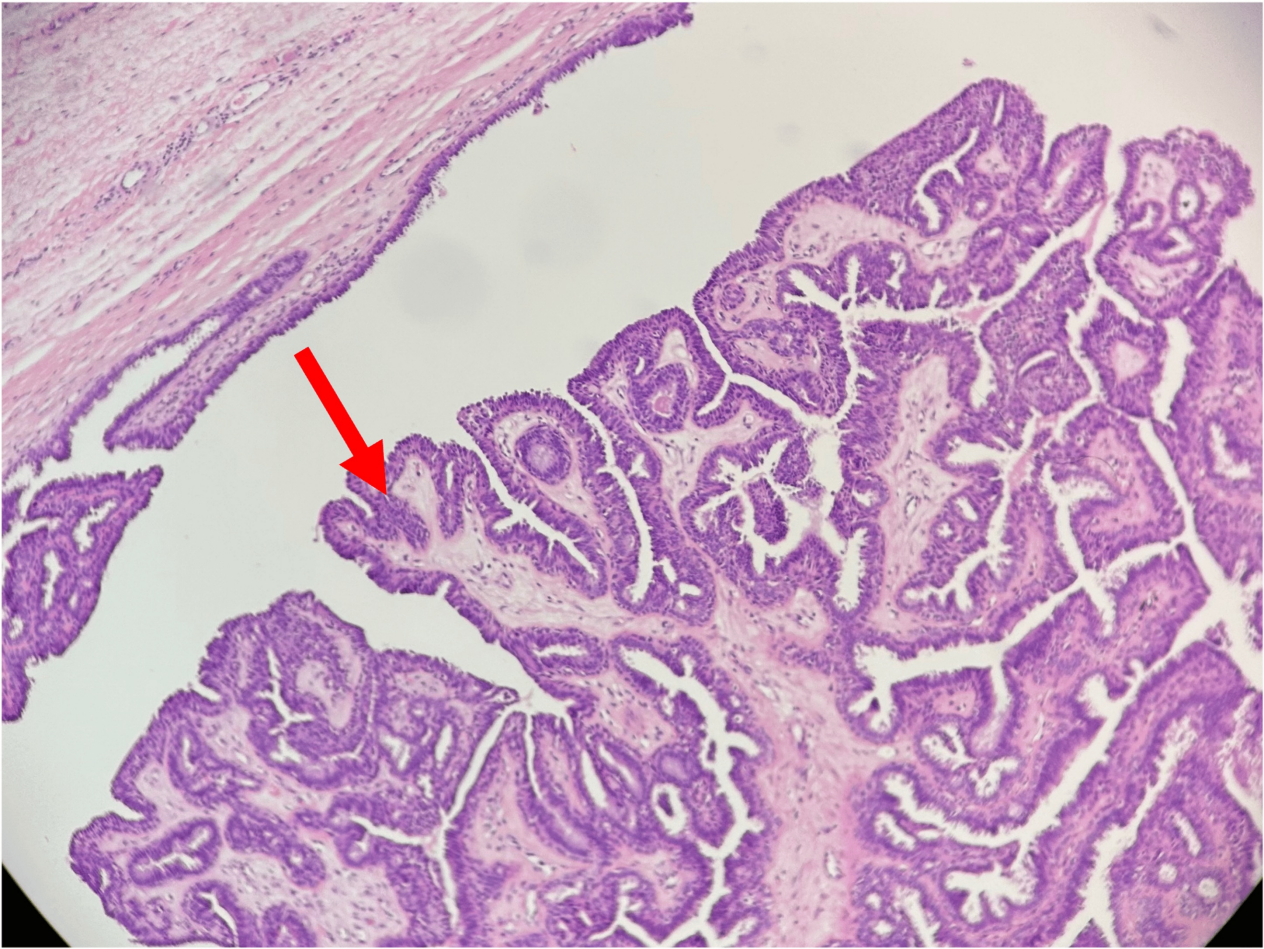
Histopathological findings High power of the polyp (×10) demonstrates a benign tumor of modified anogenital mammary-like glands (red arrow).

**Figure 3:**
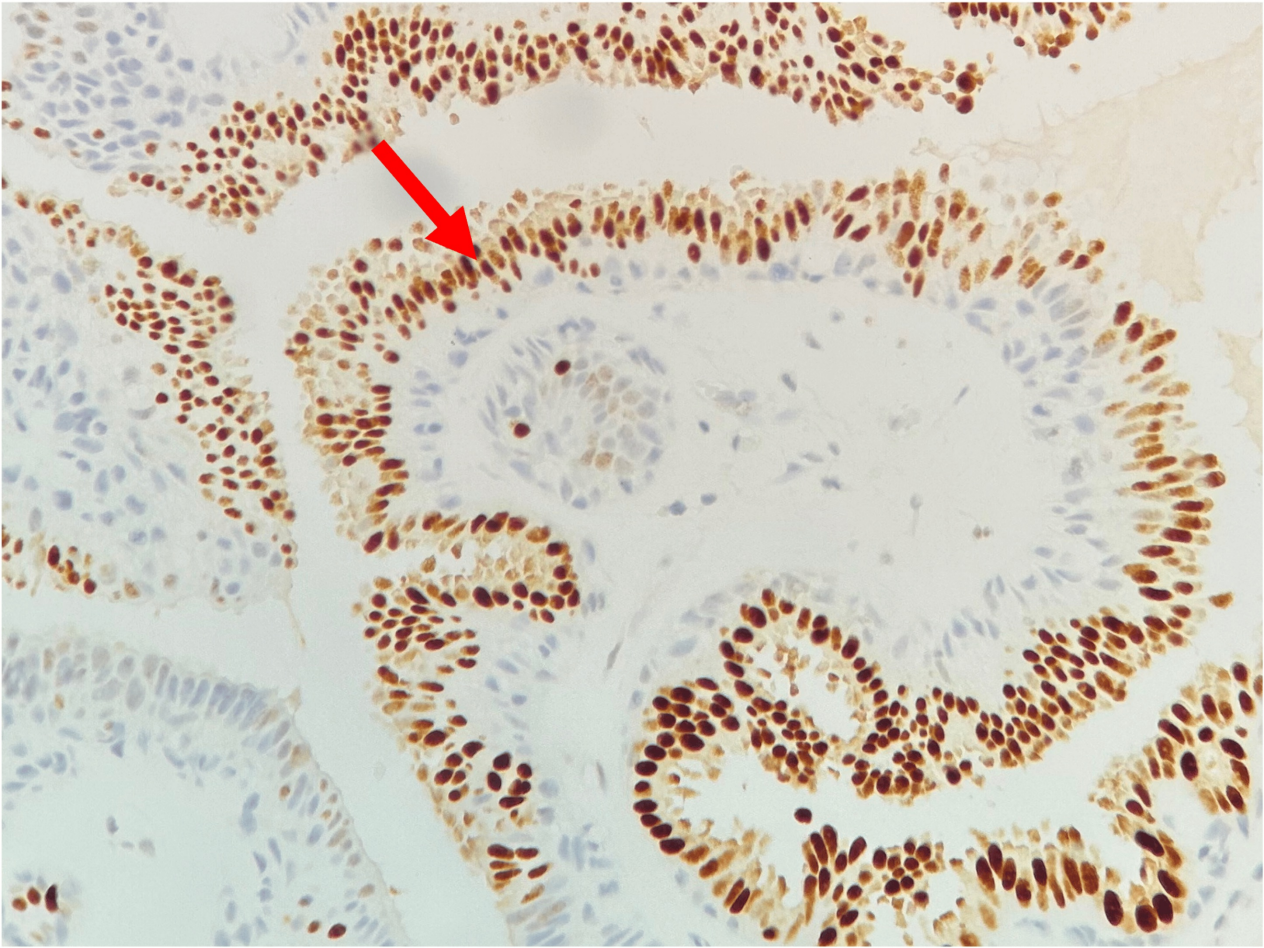
Histopathological findings An estrogen receptor positivity stain (ER stain ×20) in the luminal epithelial layer (red arrow).

**Figure 4:**
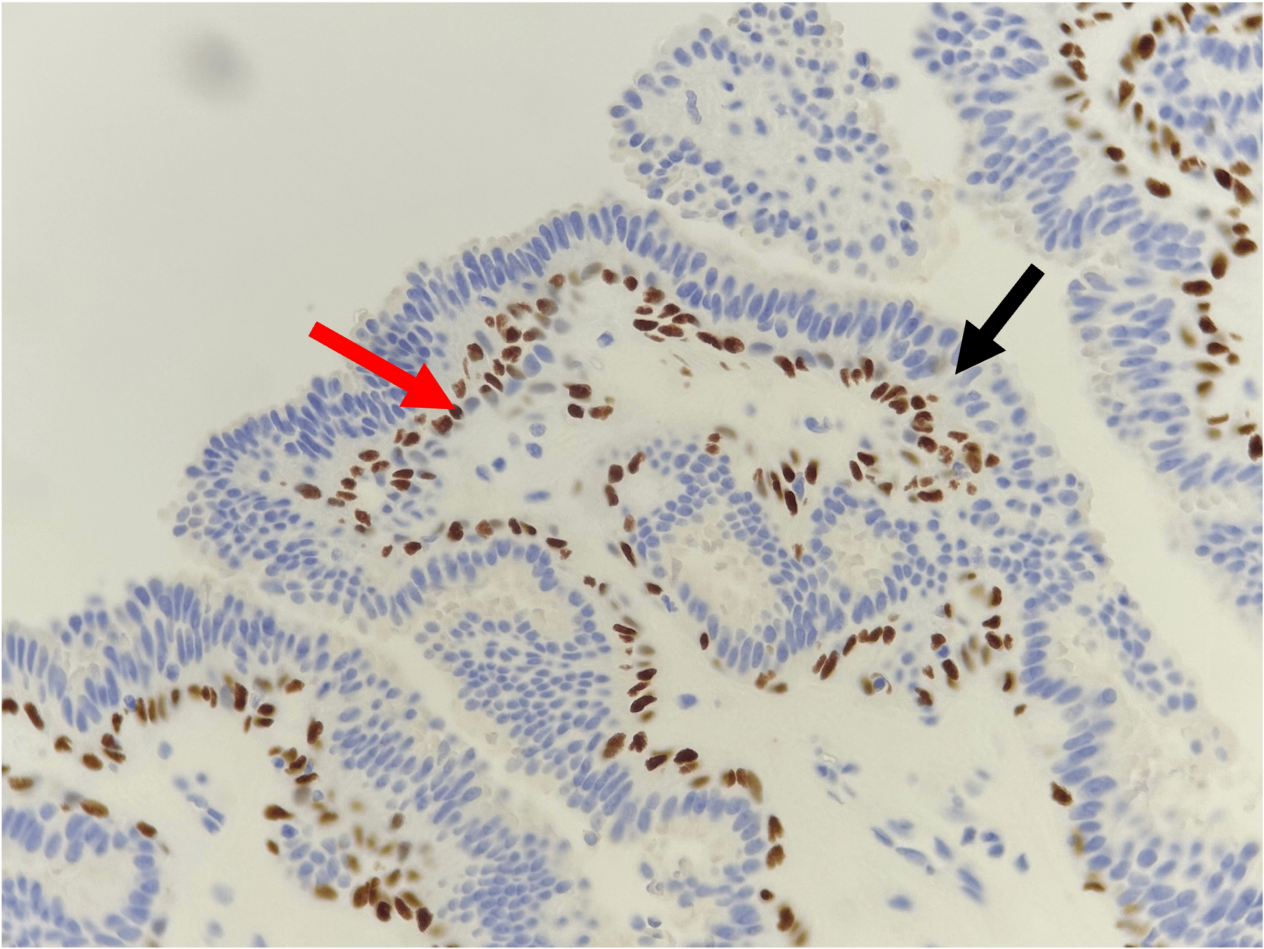
Histopathological findings A P63 stain (×20) exhibiting the presence of a myoepithelial cell layer (red arrow) and a luminal epithelial layer (black arrow).

A 54-year-old female with a past medical history of hypertension and recurrent hemorrhoids presented with worsening anorectal pain. The patient reports that for the past 30 years, she has experienced painful hemorrhoids requiring manual reduction following bowel movements, associated with bright red blood per rectum. She has had mild relief with aloe and potato suppositories. She had colonoscopies 10 years and 2 years ago, which were unremarkable. Previous anorectal exams demonstrated hemorrhoids. No biopsies were previously performed. Family history was significant for maternal breast cancer and paternal grandfather with colon cancer. On examination, there were no external hemorrhoids, fissures, fistulas, rectal prolapse, or masses, and sphincter tone was normal. No imaging was performed. Anoscopy revealed a 1 cm anal polyp, which was excised. Histopathology demonstrated polypoid ectopic breast tissue with sclerosed intraductal papilloma, apocrine metaplasia, and fibrocystic changes, without atypia or malignancy. Immunohistochemical staining was positive for ER and GATA3, with P63 and CK5/6 confirming myoepithelial cells; CDX-2 was negative. These findings are shown in Figures 1-4.

The patient returned to the office after the procedure, at which time the pathologic diagnosis was reviewed with her. Her symptoms were resolved. She was discharged from active care at that time. Regarding the maternal history of breast cancer and the paternal grandfather’s history of colon cancer, clinical vigilance was discussed with the patient to identify early signs of cancer.

## Discussion

Table 1 outlines the relevant clinical history, histologic findings, and immunohistochemical staining.

**Table 1:** Clinical, histologic, and immunohistochemical findings

	Findings
Clinical presentation	54-year-old female with long-standing history of hemorrhoids presenting with worsening anorectal pain and intermittent bright red blood per rectum
Lesion characteristics	1-cm anal polyp identified on anoscopy and surgically excised
Relevant history	Family history significant for maternal breast cancer and paternal grandfather with colon cancer
Gross pathology	Polypoid anorectal lesion without gross features of malignancy
Histologic architecture	Polypoid proliferation of anogenital mammary-like gland tissue with preserved biphasic ductal architecture
Key histologic features	Sclerosed intraductal papilloma, apocrine metaplasia, and fibrocystic changes
Cytologic atypia / malignancy	No atypia or malignant features identified
Estrogen receptor (ER)	Positive in luminal epithelial cells
GATA3	Positive, supporting mammary-type epithelial differentiation
P63	Positive in myoepithelial cell layer, confirming biphasic ductal structure
CK5/6	Positive in myoepithelial cells
CDX-2	Negative, excluding colorectal epithelial origin
Final pathologic diagnosis	Benign anogenital mammary-like gland tissue with intraductal papilloma, apocrine metaplasia, and fibrocystic change

The diagnostic course in our case highlights the importance of considering AGMLG in the differential diagnosis of anorectal masses, even when imaging or gross features are nonspecific. While most AGMLG lesions are benign, reports of malignant transformation, including adenocarcinoma, phyllodes tumor, and Paget disease, underscore the need for histologic evaluation and close follow-up when such tissue is identified. Currently, there are no formal guidelines regarding management. Surgical excision remains both diagnostic and curative for benign lesions and allows for complete pathologic assessment to rule out malignancy. In line with prior literature, excision is prudent in lesions showing atypical growth patterns, occurring in middle-aged patients, or classified as Kajava class I-IV, given their potential for neoplastic transformation [7,8].

Recent immunohistochemical analyses have helped clarify the cellular composition of anogenital mammary-like glands (AGMLG) and their resemblance to normal breast tissue. Konstantinova et al. demonstrated that AGMLGs possess a bilayered architecture composed of luminal epithelial cells and a surrounding myoepithelial layer, each exhibiting distinct yet complementary immunoprofiles [9]. The luminal cells strongly express low-molecular-weight cytokeratins (CK7, CK8, CK18, and CK19), epithelial membrane antigen (EMA), GCDFP-15, mammaglobin, MUC1, and GATA3, markers that parallel those of mammary ductal epithelium. In contrast, the basal layer shows positivity for high-molecular-weight cytokeratins (CK5/6, CK14, CK17), S100, and P63, confirming myoepithelial differentiation. Both layers demonstrate hormone receptor expression, including estrogen and progesterone receptors, supporting the concept that AGMLG are hormonally responsive structures intrinsic to the anogenital region rather than true ectopic breast remnants. These findings align with our patient’s lesion, which showed ER and GATA3 positivity along with P63 and CK5/6, confirming the biphasic ductal pattern typical of AGMLG. The absence of CDX2 staining excluded colorectal origin, reinforcing that the excised anal polyp represented mammary-like gland tissue exhibiting benign proliferative changes [9].

In addition to immunohistochemical staining, colorectal adenomas are distinct from AGMLGs in origin and morphology. They are precancerous polyps arising from the colorectal mucosa, while AGMLGs are normal glands found in the skin located in the perianal or vulvar region. They are classified based on histological architecture into tubular (resembling round tube-shaped glands), villous (characterized by long finger-like projections), or mixed (referred to as tubulovillous). Immunohistochemistry of colorectal adenomas expresses CK20 and CDX2. Another differential diagnosis includes hidradenoma papilliferum (HP). It is a benign tumor arising from AGMLGs, but it is clearly distinguishable based on its characteristic maze-like/cystic morphology with papillary fronds showing dual epithelial layers (columnar/myoepithelial) and apical secretory buds, often in the interlabial sulcus. The lack of epidermal connection differentiates it from normal glands or other benign MLG tumors such as syringocystadenoma papilliferum [10].

Medical guidelines categorize breast cancer risk levels based on the lifetime risk of developing breast cancer. Average risk is less than 15%. Intermediate risk is 15% to 19%. High risk is 20% or greater [11]. Based on the National Cancer Institute’s breast cancer risk assessment tool, the patient’s five-year risk of breast cancer is 2.1%. The U.S. Preventive Services Task Force (USPSTF) recommends that all women undergo a screening mammogram every two years, starting at age 40 and continuing through age 74, for those with average risk, family history, or dense breasts, but not for high-risk individuals, such as BRCA carriers [12]. The National Comprehensive Cancer Network (NCCN) recommends BRCA testing for breast cancer diagnosis at age 50 or younger, triple-negative breast cancer diagnosis, multiple primary breast cancers, any personal history of ovarian, fallopian tube, primary peritoneal cancer, pancreatic cancer, or high-risk metastatic prostate cancer, male breast cancer, a first-degree relative with a known mutation, two or more relatives in the same family diagnosed with breast cancer, or Ashkenazi Jewish heritage [13]. Based on the USPSTF and NCCN recommendations, the patient’s lifetime breast cancer risk is average, so routine breast cancer screening and patient education concerning the risk of recurrence are advised; BRCA testing is not indicated. Benign lesions can be conservatively excised or monitored based on symptoms and patient preference.

Finally, our report highlights the need for ongoing awareness and research regarding AGMLGs. Larger case series could help clarify the prevalence, clinical course, and risk factors for neoplastic change in these lesions. Molecular studies may further elucidate the pathogenesis of intraductal papillomas or other neoplastic transformations within anogenital mammary-like glands. It is worth noting that AGMLGs are often confused with hidradenoma papilliferum because of the similarities in morphology and immunohistochemistry staining. Clinicians should remain vigilant for these rare entities and consider histologic evaluation in any unusual anorectal mass to ensure timely diagnosis and management.

## Conclusions

This case highlights the importance of recognizing AGMLGs as orthotopic, hormonally responsive glands capable of mimicking lesions identical to counterparts in the female breast. This underscores that confirmation of diagnosis with histologic evaluation is crucial for diagnosing and managing benign and potentially malignant pathologies. Our patient’s findings of intraductal papilloma with apocrine metaplasia and fibrocystic change highlight the benign potential of AGMLG lesions but also the need for awareness of their possible malignant transformation. Surgical excision remains the mainstay of both diagnosis and treatment. Continued reporting and study of such cases will enhance understanding of their behavior, guide management, and prevent misdiagnosis of these uncommon but clinically significant entities.
